# Accumulation of Potentially Toxic Elements in Mosses Collected in the Republic of Moldova

**DOI:** 10.3390/plants10030471

**Published:** 2021-03-02

**Authors:** Inga Zinicovscaia, Constantin Hramco, Omari Chaligava, Nikita Yushin, Dmitrii Grozdov, Konstantin Vergel, Gheorghe Duca

**Affiliations:** 1 Joint Institute for Nuclear Research, Joliot-Curie 6, 141980 Dubna, Russia; costea.edinets@mail.ru (C.H.); omar.chaligava@ens.tsu.edu.ge (O.C.); ynik_62@mail.ru (N.Y.); dsgrozdov@rambler.ru (D.G.); verkn@mail.ru (K.V.); 2 Horia Hulubei National Institute for R&D in Physics and Nuclear Engineering, 30 Reactorului Str., MG-6, 041713 Bucharest-Magurele, Romania; 3 Institute of Chemistry, 2002 Chisinau, Moldova; ggduca@gmail.com; 4 Department, Ivane Javakhishvili Tbilisi State University, Chavchavadze ave. 3, Tbilisi, GA 0179, USA

**Keywords:** moss biomonitoring, Republic of Moldova, chemical elements, neutron activation analysis, pollution indices

## Abstract

For the second time, the moss biomonitoring technique was applied to evaluate the deposition of potentially toxic elements in the Republic of Moldova. The study was performed in the framework of the International Cooperative Program on Effects of Air Pollution on Natural Vegetation and Crops. Moss *Hypnum cupressiforme* Hedw. samples were collected in May 2020 from 41 sampling sites distributed over the entire territory of the country. The mass fractions of 35 elements (Na, Mg, Al, Cl, K, Ca, Sc, Ti, V, Cr, Mn, Fe, Co, Ni, Cu, Zn, As, Br, Se, Rb, Sr, Sb, Cs, Ba, Cd, La, Ce, Sm, Eu, Tb, Hf, Ta, Th, Pb, and U) were determined using neutron activation analysis and atomic absorption spectrometry. Comparing with 2015/2016 moss survey data, significant differences in the mass fractions of Cr, As, Se, Br, Sr, Sb, Cd, Pb, and Cu were found. Main air pollution sources (natural processes, transport, industry, agriculture, mining) were identified and characterized using factor and correlation analyses. GIS maps were built to point out the zones with the highest element mass fractions and to relate this to the known sources of contamination. Contamination factor, geo-accumulation index, pollution load index, and potential ecological risk index were calculated to assess the air pollution levels in the country. According to the calculated values, Moldova can be characterized as unpolluted to moderately polluted, with low potential ecological risk related to the degree of atmospheric deposition of potentially toxic elements. The cities of Chisinau and Balti were determined to experience particular environmental stress and are considered moderately polluted.

## 1. Introduction

Toxic elements, regardless of the source of their provenance, natural, or anthropogenic, increase the pressure on the environment and are extremely harmful to human health [1]. Pollution of the atmosphere with potentially toxic elements has attracted great environmental concern since it was proven that this type of pollution can provoke different respiratory illnesses in humans [2]. Monitoring of potentially toxic air pollutants has a significant role in the understanding of their spatial and temporal distribution and elaboration of planned actions to reduce their harmful effects [3].

Monitoring technique using living organisms is a reliable and economically viable procedure for estimating the degree of environmental pollution [4]. Suitable biological organisms (mosses, lichens) are used as biomonitors and indicators of atmospheric deposition of potentially toxic elements [5,6,7,8]. Among those organisms, mosses have been widely used as an indicator of atmospheric pollution by toxic elements, as reported by various researchers [1,3,4,9,10,11,12,13,14]. Due to their anatomical structure (lack of root system), ubiquity, high surface-to-volume ratio, and ion-exchange capacity mosses effectively accumulate atmospheric pollutants, especially potentially toxic elements at mass fractions above their physiological needs [1,2,4]. In addition, due to the slow growth rate, moss growth segments can be used as an estimate of the integrated exposure to toxic metals over longer periods, and not just the current state at the time of collection, which is particularly important in areas where levels of emissions of potentially toxic elements change rapidly [15].

The majority of the research using moss as biomonitors is performed in the framework of the United Nations Economic Commission for Europe International Cooperative Program on Effects of Air Pollution on Natural Vegetation and Crops (UNECE ICP Vegetation), which was established in 1990 and covers most European countries. Twenty-one European countries contributed to the first campaign of the project [16]. Since 1990, moss surveys have been performed every five years and the number of countries involved in the program continuously increases [17]. For the first time, the Republic of Moldova joined the ICP Vegetation program in the 2015/2016 moss survey, when samples were collected throughout the country. Based on the obtained data, the cities of Chisinau and Balti were determined to experience particular environmental stress. The level of air pollution in the country varied from moderate to severe and the main emission sources were identified as thermoelectric plants (V, U, Sb, As), transport (Pb), and industry (Fe, Cr, Zn) [14].

The purpose of the present study was to investigate the deposition of potentially toxic elements in the Republic of Moldova by using the moss biomonitoring technique in order to reveal changes in element accumulation in comparison with previous moss survey.

## 2. Results and Discussion

Results of the descriptive statistics of 35 elements determined in 41 samples are shown in Table 1.

For all determined elements, values of the coefficients of variation (CV) were in the 25–75% range, indicating moderate variations in the mass fraction. It is suggested that for each element a moderate variation reflects similar contamination levels throughout the monitored region and indicates the stability of the content in moss tissues [18]. Zhao et al. [19] showed that particle grain size is an important indicator for the variability in physical characteristics and pollutants composition and the CV values increased greatly as the particle size increased. Thus, low values of CV may be indicative of very small particles, while high CV values may represent coarse particles.

### 2.1. Comparison of the Obtained Values with Data from the Previous Moss Survey

In Table 1 the median values ± MAD of the content of elements determined in the present study and the 2015/2016 moss survey are given as well. Differences in the measurements of individual elements between the two sampling campaigns were investigated using the Wilcoxon test. According to the test, there were no significant differences (*p* > 0.05) for the main part of the elements in samples collected in 2015 and 2020. Significant differences (*p* < 0.05) between the mean mass fractions were revealed for Cr, As, Se, Br, Sb, Cd, Pb, and Cu. The median mass fractions of these elements were lower in 2020. The differences between the two moss surveys may reflect changes in the bioavailability of the elements resulting from wet and dry deposition rates during 2020 and 2015 [11].

Since data for the 2020/2022 moss survey for neighboring countries (Ukraine, Romania, Bulgaria, Poland, Belarus, and Russia) have not been published yet, the median values obtained in the present study were compared with data from the European Moss Atlas, related to the 2015/2016 moss survey [17]. Comparison of the median values for each element showed that among the compared countries, the content of As, Al, Ni, V, Cr, and Fe are the highest in mosses collected in Moldova, except Romania (Table 2). The content of Cd in Moldova was the lowest and of Pb—Among the lowest. In comparison with Romania, Russia, and Poland, Moldova has lower values for Zn.

The sources of high mass fractions of Al, Fe, Ni, V, and As in moss samples in Moldova, besides anthropogenic sources, are soil particles, which usually increase mass fractions of these elements in moss in zones with a dry climate. The relatively dry weather with low rainfall and large areas of cropland in Moldova substantially contribute to the distribution of mineral particles and subsequent accumulation by moss species [15]. According to Kłos et al. [20], two different mechanisms control the translocation of metals from soil to epigeal mosses. The first mechanism consists of transporting metals with dust uplifted from the soil, while the second one employs diffusion of metal cations through aqueous solutions wetting the mosses. At the same time, in rainy regions, the efficiency of metal uptake by mosses is very low and the heavier the rain, the less metal uptake efficiency is obtained [21].

### 2.2. Association of Chemical Elements

Factor and correlation analyses were applied to provide a multivariate view of the distribution of the elements and to reveal the origin of pollution sources for the elements of interest. The matrix of rotated factor loadings is given in Table 3.

**Figure 1:**
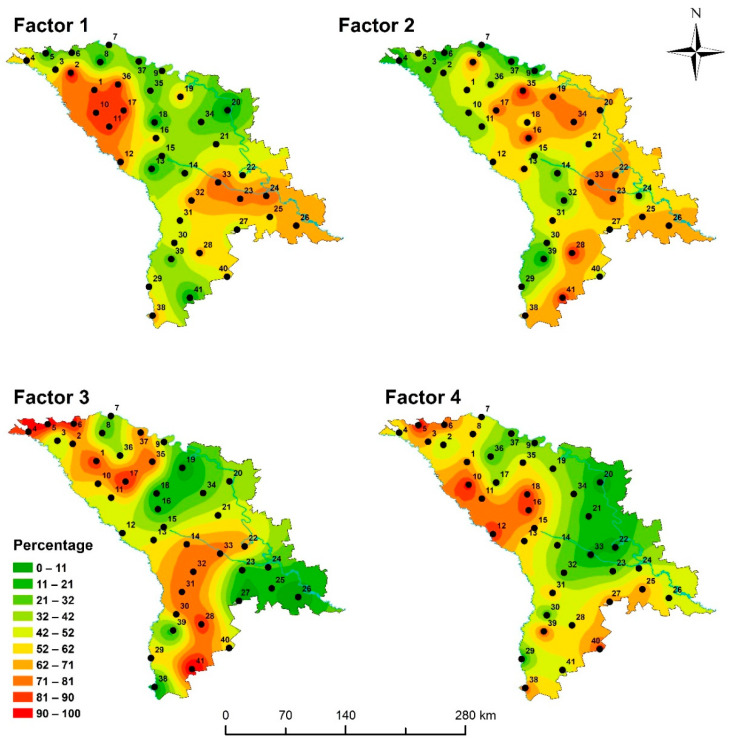
Map showing spatial distributions of Factors 1–4.

Four factors were identified, including 84% of the variability of the treated elements (a Scree plot is given in Figure S1). Factor 1 (Na-Al-Sc-Ti-Cr-Fe-Co-Ni-As-Rb-Sb-Cs-Th-U) is the strongest factor representing 47% of the total variability. The presence of Al, Sc, Ti, and Th in this group confirms the terrigenous origin of these elements. Since Moldova is characterized by a dry climate with a low amount of precipitation, moss contamination with windblown dust and mineral particles takes place due to soil erosion. At the same time, elements such as Cr, Ni, and As can have an anthropogenic origin. The highest values of the factor scores in the Balti and Chisinau areas confirm this assumption (Figure 1). In 2015, the highest mass fractions of the Factor 1 elements were determined near Chisinau, Balti, Rezina, and Stefan Voda. In 2020, the highest mass fractions of the elements which represent Factor 1 were determined in the Balti and Chisinau areas [14].

With factor 2 (contribution 14%) are associated the elements Mg, Ca, and Sr. The main source of these elements may be mining activities. Currently, there are 411 prospected deposits of 17 types of mineral resources in the Republic of Moldova. About 900 quarries are used for mineral extraction. The main extracted minerals are limestone, clay, sand, granite, gypsum [22]. The highest mass fractions of the aforementioned group of elements were found in moss samples collected in areas where the quarries for limestone and gypsum extraction are operated.

Factor 3 is the third strongest factor, with 12% of the total variability and associated elements Cl, K, Br, and Cu, suggesting inputs by agricultural activities. Considerable amounts of Br and Cl are used as fungicides and as components of K fertilizers [23]. In our previous study, high content of Br was determined in Moldavian soil [24]. Copper is a component of micronutrient fertilizers and copper-based fungicides. Copper fungicides are widely used in viticulture [23,24,25]. Factor 4 (Zn-Sb-Cd-Pb) is anthropogenic and represents 11% of the total variability of the dataset. It includes elements that are considered indicators of emission from fossil fuel combustion processes, including vehicle exhaust. According to the distribution map of the factor scores (Figure 1), high mass fractions of these elements are present in the north and north-east parts of the country.

**Figure 2:**
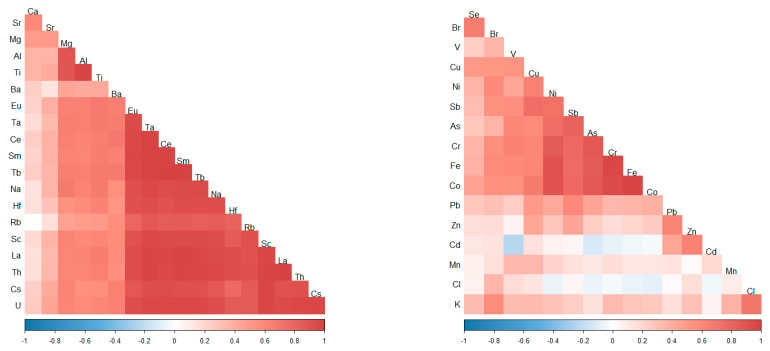
Pearson correlation coefficient between element content in mosses in Moldova: matrix 1 (**left**) and matrix 2 (**right**).

Correlation analysis was performed to test the relationship between the elements (Figure 2). For better visibility, the matrix of correlation coefficients was divided into two parts. The first matrix presents a correlation of 19 selected naturally distributed elements, and the second one of 16 elements, which can originate from natural as well as anthropogenic sources. The results of the correlation analysis repeat very well the results of the factor analysis. A strong positive correlation of elements characteristic for soil was observed. On the second matrix, K correlated well with Cl, Cd with Zn, Zn with Pb, Pb with Sb and Cu. Cobalt, Fe, and Cr are positively correlated with one another as well as with Ni, As, V, and Cu. Arsenic, Sb, Ni, and Cu positively correlate with each other, as well as with V and Br. Bromine shows a positive correlation with Se.

### 2.3. Pollution Assessment of the Examined Moss Samples

In this study, to assess the degree of air pollution in Moldova Contamination factor (CF) [26] and Pollution load index (PLI) [27] were calculated for elements which are listed as priority pollutants to control by the European Economic Area report [28]. The values of the indices were calculated for the whole country and separately for Chisinau and Balti. The results are presented in Table 4.

The mean CF values of Cd below 1.0 indicate minimal anthropogenic effects with uncontaminated levels in the entire country, Chisinau, and Balti. The mean CF values of Pb and Cu were less than 1.0 for the entire country but greater than 1.0 in Chisinau and Balti, indicating slight pollution in the cities. In Moldova, CF levels from 1 to 3 indicating moderate contamination levels were obtained for V, Cr, Fe, As, Zn, Sb, and U. In Chisinau and Balti, the CF values of Zn indicate suspected pollution, and those of Cu, V, Cr, Fe, As, Sb, and U—slight to moderate pollution. The mean value of PLI for the entire country indicates unpolluted to moderately polluted conditions and for both cities, Chisinau and Balti, the results show moderately polluted conditions.

The PER values reflect the sensitivity of various biological communities to toxic substances and represent the potential ecological risks posed by hazardous elements [19]. The ecological risk values for single metals decreased in the following order As > Cd > Cu > Pb > Cr > Zn and the mean PER values for these metals were 16.3, 16, 4.7, 3.6, 2.6, and 1.0, respectively. The mean ecological risk value of 44.1 indicates low potential ecological risk.

## 3. Materials and Methods

### 3.1. Studied Area

The Republic of Moldova (Moldova) with a surface area of 33,800 km^2^ and a population of 3,546,000 is situated in Eastern Europe. Moldova is a landlocked country and shares borders with Ukraine and Romania. The country extends 350 km from North to South and 120 km from West to East. The capital of Moldova, the city Chisinau with a population of 700,000, is located in the central part of the country. The country is relatively low-lying and hilly, with semi-arid steppe plains in the south. The climate is continental, with relatively mild winters characterized by an average daily temperature between −5 °C and −3 °C and little snow and warm summers with limited rainfall. The average annual precipitation varies between 617 mm in the North and 546 mm in the South.

In Moldova, air quality monitoring is performed by the State Hydrometeorological Service, which has a network of 17 stations, located in Chisinau, Balti, Tiraspol, Ribnita, and Bender. At present, approximately 4000 stationary sources of air pollution are registered in the Republic of Moldova, including three power and heat generation facilities, 40 regional, 28 inter-regional, and 1639 local boiler houses, 530 gasoline and gas stations, 24 big fuel storage sites [22]. Another important local source of air pollution is transport.

### 3.2. Sampling

**Figure 3:**
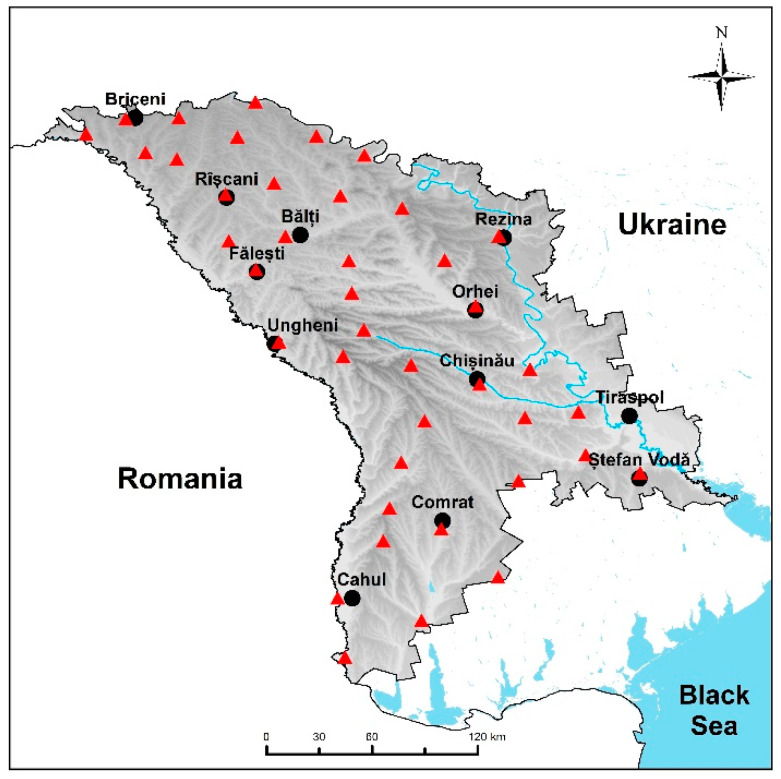
Location of sampling points. More details about sampling sites can be found in Table S1.

Since *Hypnum cupressiforme* is the predominant moss species in Moldova, samples only of this species were collected during two performed moss survey campaigns. In April 2020, moss samples were collected at 41 sampling sites evenly distributed over the territory of Moldova (Figure 3). The samples were collected at the same 33 locations as in 2015 and 8 new sampling sites were added, which cover the South part of the country. Moss samples were collected following the manual of the International Cooperative Program on Effects of Air Pollution on Natural Vegetation and Crops [29].

According to the manual, each country should aim to collect at least 1.5 moss samples/1000 km^2^. If this is not feasible, a sampling density of at least two moss sample sites per grid (50 km × 50 km) is recommended. In our case, moss samples were collected in a grid with a spacing of approximately 30 km × 30 km. More dense moss sampling was not possible due to the absence of mosses. Moss samples were collected on the ground or surface of decaying stumps at least 3 m away from the nearest projected tree canopy. Samples were collected at a distance of least 300 m away from villages and industries, and at least 100 m from smaller roads. The main criteria regarding the sampling were: about 0.5 kg of fresh moss was collected at each sampling point, consisting of five to ten sub-samples of the same moss species. A separate set of polyethylene gloves was used for the collection of each sample. Collected samples were stored in air-permeable bags.

### 3.3. Sample Preparation

The collected moss samples were cleaned of foreign material, and the upper 3–4 cm of the green and green-brown shoots from the top of the moss, which represents the last 3 years of growth, was separated and dried at 40 °C to constant weight. For neutron activation analysis (NAA) moss samples of about 0.3 g were pelletized and packed in polyethylene foil bags for short-term irradiation and in aluminum cups for long-term irradiation. 

For atomic-absorption analysis (AAS) approximately 0.2 g of moss was placed in a Teflon vessel and treated with 2 mL of concentrated nitric acid and 1 mL of hydrogen peroxide. The Teflon vessels were put into a microwave digestion system (Mars; CEM, USA) for complete digestion. Digestion was performed in two steps: (1) ramp: temperature 180 °C, time 15 min, power 400 W, and pressure 20 bar; (2) hold: temperature 160 °C, time 10 min, power 400 W, and pressure 20 bar. Digests were quantitatively transferred to 100-mL calibrated flasks and made up to the volume with bi-distilled water. All of the reagents used for this study were of analytical grade: nitric acid; trace pure (Merck, Germany); hydrogen peroxide, p.a. (Merck, Germany); and bi-distilled water.

### 3.4. Analysis

#### 3.4.1. Neutron Activation Analysis

The content, in mg/kg dry weight, of 32 elements (Na, Mg, Al, Cl, K, Ca, Sc, Ti, V, Cr, Mn, Fe, Co, Ni, Zn, As, Br, Se, Rb, Sr, Sb, Cs, Ba, La, Ce, Sm, Eu, Tb, Hf, Ta, Th, and U) in moss samples was determined by neutron activation analysis at the IBR-2 reactor (JINR, Dubna, Russia). To determine elements with short-lived isotopes (Cl, V, Ti, Mg, Al, Ca, and Mn) samples were irradiated for 3 min at a thermal neutron flux of 1.6 × 10^12^ n cm^−2^ s^−1^ and measured for 15 min. To determine elements with long-lived isotopes: Na, K, Sc, Cr, Fe, Co, Ni, Zn, As, Br, Se, Rb, Sr, Sb, Cs, Ba, La, Ce, Sm, Eu, Tb, Hf, Ta, Th, and U samples were irradiated for 4 days at a neutron flux 3.31 × 10^11^ n cm^−2^ s^−1^, re-packed, and measured twice using HP-Ge detectors after 4 and 20 days of decay, respectively. Gamma spectra processing and determination of element mass fractions were performed using Genie 2000 and software developed in FLNP JINR [30].

#### 3.4.2. Atomic Absorption Spectrometry

The content of three elements, Cd, Cu, and Pb, in the moss samples was determined by using iCE 3400 AAS Atomic Absorption Spectrometer with electrothermal (graphite furnace) atomization (Thermo Fisher Scientific, Waltham, MA, USA). The calibration solutions were prepared from AAS standard solutions with metal ion concentrations of 1 g/L (Merck, Germany). 

#### 3.4.3. Quality Control

The quality control of NAA results was ensured by simultaneous analysis of the examined samples and the following standard reference materials: NIST SRM 1547 (Peach leaves), NIST SRM 1575a (Trace Elements in Pine Needles), NIST SRM 2709 (San Joaquin Soil), NIST SRM 2711 (Montana Soil), and IC-INCT-OBTL-5 (Oriental Basma tobacco leaves). The use of standards of different matrices allowed to expand the number of elements with certified values determined in moss the samples, since the standard material for vegetation contains only a limited number of certified values. Chemical matrix effects, known to be significant sources of error in other types of instrumental chemical analysis, are insignificant in NAA. The use of reference materials with a matrix different from the analyzed samples in NAA is explained by the insignificant matrix effect in the case of small samples (size and weight) [31].

Quality control of the AAS results was ensured using the NIST SRM 1570a (Trace Elements in Spinach Leaves) and NIST SRM 1575a (Trace Elements in Pine Needles). The difference between the measured and certified values did not exceed 15% for NAA and 3% for AAS.

#### 3.4.4. Statistical Analysis and Mapping

Processing of the obtained data was performed using Excel (Microsoft, Redmond, Washington, DC, USA) and IBM SPSS software (IBM, Armonk, New York, NY, USA). Descriptive statistics for the determined elements in samples from 41 locations were calculated (Table 1). The Wilcoxon signed-rank test [32] was applied to investigate differences between the values obtained in the 2015 and 2020 moss surveys. To discover associations of chemical elements and decrease the number of variables for the obtained data, factor analysis, and correlation analysis were used. Since many statistical techniques including factor analysis are sensitive to non-normally distributed data, the Box-Cox transformation was performed. The ArcGIS software (Esri, Redlands, CA, USA) was used to build maps showing the spatial distributions of elements using the radial basis functions method.

### 3.5. Pollution Indices

The contamination factor CF is defined as the ratio between the mass fraction of an element in moss samples and its background level in moss [26]:

(1) $$CF=\frac{C_{m}}{C_{b}}$$

where *C_m_* is the content of a certain metal at any collection site and *C_b_* is the background level for the same metal. *CF* < 1 implies no contamination; 1–2—suspected; 2–3.5—slight; 3.5–8—moderate; 8–27—severe; and >27—extreme [26]. In a study [33], the background concentrations of heavy metals were considered to be those obtained by measuring the levels of different elements in areas assumed to be unaffected by human activity. In our study, as background, were considered values obtained for mosses collected in the area of the Capriana national reserve.

The PLI represents the nth order geometric mean of the entire set of CF values [27]:

(2) $$\mathrm{PLI}=\sqrt[{n}]{\prod_{i=1}^{n}C_{F,i}},$$

where n is the total number of contaminating elements. 

According to the contamination degree, the PLI data were classified as unpolluted (PLI < 1), unpolluted to moderately polluted (1 < PLI < 2), moderately polluted (2 < PLI < 3), moderately to highly polluted (3 < PLI < 4), highly polluted (4 < PLI < 5), or very highly polluted (PLI < 5) [27].

Potential ecological risk index, RI, can be applied for ecological risk assessments of chemical elements in moss samples and is defined by formulas:

(3) $$\mathrm{RI}=\sum {\mathrm{PER}}_{f}^{i}$$

(4) $${\mathrm{PER}}_{f}^{i}=C_{f}^{i}\times T_{f}^{i}$$

where ${\mathrm{PER}}_{f}^{i}\;$is the potential ecological risk index of each element $C_{f}^{i}\;$is the contamination factor, and $T_{f}^{i}$ is the “toxic-response” coefficient for the given single metal. The toxic response factors for Cr, Ni, Cu, As, Cd, Zn, and Pb are 2, 6, 5, 10, 30, 1, and 5, respectively [34]. The ecological risk was classified in four groups: RI < 150—low ecological risk; 150 ≤ RI < 300—moderate ecological risk; 300 ≤ RI < 600—considerable ecological risk; RI ≥ 600—very high ecological risk [34].

## 4. Conclusions

- During the second moss survey study in Moldova, the mass fractions of 35 elements were determined using NAA and AAS. The mass fractions of the determined elements varied in a wide range and the highest concentrations were determined in urban areas, mainly in Chisinau and Balti.
- Comparison of the obtained results with data from the previous moss survey revealed a significant decrease of the mass fractions of Cr, As, Se, Br, Sb, Cd, Pb, and Cu in the present moss survey.
- Compared with moss survey results from neighboring countries, the mass fractions of the elements As, Al, Ni, V, Cr, and Fe were the highest in samples collected in Moldova, while of Cd and Pb they were among the lowest.
- According to factor analysis to the main air pollution sources ascertained during the 2015/2016 moss survey in Moldova, namely, transport, industrial activity, and thermal power plants, were added mining and industrial activities.
- Contamination factor and Pollution load index values revealed unpolluted to moderately polluted conditions. The Balti and Chisinau municipalities were found to be the most contaminated. It was determined that Cr, Ni, Cu, As, Cd, Zn, and Pb pose a low potential ecological risk.

## Figures and Tables

**Table 1:** Results of the descriptive statistics of measurements for moss samples (in mg kg^−1^) collected in 2020 and comparison of the results from the present study with values from 2015/2016 moss survey (mg/kg).

	2020/2022					2015/2016	
	Range	Md ± MAD ^&^	Q1	Q3	Percentile 90	Md ± MAD	Analytical Technique
Al	1280–11,700	3400 ± 1200	2515	4780	8784	3120 ± 1100	NAA
As *	0.31–2.03	0.77 ± 0.23	0.56	1.06	1.35	0.85 ± 0.27	NAA
Ba	24–117	50 ± 15	40.8	71.5	84	60 ± 24	NAA
Br *	1.07–7.6	3.2 ± 1.6	1.78	4	5.72	4.7 ± 1.0	NAA
Ca	5740–17,200	9300 ± 1500	8165	11,050	16,200	9900 ± 1100	NAA
Cd *	0.06–0.56	0.12 ± 0.04	0.078	0.16	0.25	0.39 ± 0.08	AAS
Ce	1.83–16	4.6 ± 1.8	3.47	7.7	9.42	4.4 ± 1.7	NAA
Cl	23–453	110 ± 30	67.5	139	174	100 ± 40	NAA
Co	0.4–3.24	0.98 ± 0.29	0.7	1.32	1.96	0.79 ± 0.29	NAA
Cr *	3.2–21.3	5.5 ± 1.06	4.4	8.95	11.7	7.2 ± 3.1	NAA
Cs	0.18–1.5	0.42 ± 0.13	0.29	0.6	0.79	0.33 ± 0.14	NAA
Cu *	5.7–22.2	8.7 ± 1.0	7.12	9.42	11.7	15± 3.0	AAS
Eu	0.02–0.27	0.08 ± 0.04	0.058	0.12	0.15	0.08 ± 0.04	NAA
Fe	951–7810	2200 ± 600	1740	3125	4524	2100 ± 900	NAA
Hf	0.14–1.83	0.56 ± 0.19	0.41	0.86	1.15	0.45 ± 0.22	NAA
K	4170–12,100	7250 ± 1000	5535	7830	9896	7100 ± 1500	NAA
La	0.78–8.1	2.3 ± 0.6	1.71	3.05	4.58	2.1 ± 0.8	NAA
Mn	41–335	90 ± 30	73.5	148	199	120 ± 50	NAA
Na	119–965	400 ± 120	248	506	670	308 ± 122	NAA
Ni	2.2–14.3	4.1 ± 1.0	3.27	5.4	7.74	4.7± 2.0	NAA
Pb *	1.56–8.82	3.1 ± 0.4	2.76	3.74	5.12	12 ± 2.5	AAS
Rb	3.2–26.6	9.9 ± 2.1	7.5	12.1	17.8	9.8 ± 3.9	NAA
Sb *	0.09–0.85	0.19 ± 0.04	0.15	0.26	0.41	0.25 ± 0.06	NAA
Sc	0.28–2.84	0.76 ± 0.24	0.59	1.07	1.61	0.69 ± 0.31	NAA
Se *	0.11–0.43	0.23 ± 0.04	0.18	0.26	0.29	0.32 ± 0.05	NAA
Sm	0.15–1.3	0.39 ± 0.14	0.28	0.57	0.77	0.31 ± 0.13	NAA
Sr	26.5–107	50 ± 15	38.3	68.5	92	40 ± 10	NAA
Ta	0.02–0.21	0.06 ± 0.02	0.046	0.095	0.12	0.06 ± 0.03	NAA
Tb	0.02–0.15	0.04 ± 0.01	0.032	0.068	0.087	0.05 ± 0.02	NAA
Th	0.23–2.5	0.73 ± 0.22	0.53	1	1.49	0.65 ± 0.27	NAA
Ti	80–1020	290 ± 80	207.5	379	668	230 ± 110	NAA
U	0.08–0.62	0.21 ± 0.07	0.16	0.29	0.35	0.22 ± 0.09	NAA
V	2.4–18.8	5.4 ± 1.6	3.9	8.2	13.7	5.5 ± 2.3	NAA
Zn	25–86	39 ± 7	32.3	47	70.4	37.2 ± 8.5	NAA

^&^ Md—median, MAD—Median Absolute Deviation, Q1 and Q3—the first and the third quartile, * -significantly different

**Table 2:** Comparison of the median values for selected elements in Moldova with corresponding data from neighboring countries * [18], in mg/kg.

	Al	As	Cd	Cr	Cu	Fe	Ni	Pb	Sb	V	Zn
Moldova	3400	0.7	0.1	5.4	8.7	2200	4.1	3.1	0.2	5.4	40
Belarus	595	0.23	0.39		5.52	392	1.3	2.18	0.096	0.95	35
Bulgaria	2290	0.44	0.12	2.76	7.28	1125	2.21	10.7	0.11	3.81	28.1
Poland	967	0.38	0.21	2.22	7.6	535	2.94	4.98	0.2	1.59	50.6
Romania	2895	1.08	0.27	4.72	5.77	1535	3.11	4.2	0.2	4.32	40.1
Russia	1450	0.49	0.28	4.13	6.03	925	2.55	0.81	0.2	2.65	43.1
Ukraine	938	0.7	0.31	3.65	10.4	700	2.89	3.81	0.19	2.52	33.7

* for neighboring countries, the results of 2015/2016 moss survey are presented.

**Table 3:** Matrix of rotated factor loadings (Box-Cox transformation used).

Element	Factor 1	Factor 2	Factor 3	Factor 4	Communality, %
Na	0.93	0.18	0.14	−0.02	98
Mg	0.53	0.68	0.26	0.17	95
Al	0.60	0.54	0.41	0.27	100
Cl	−0.18	0.18	0.82	−0.07	82
K	0.08	0.03	0.86	−0.15	85
Ca	0.02	−0.81	−0.06	0.38	76
Sc	0.97	0.14	0.12	−0.07	100
Ti	0.62	0.55	0.36	0.25	98
V	0.58	0.56	0.42	0.20	100
Cr	−0.91	−0.21	−0.09	0.11	94
Fe	−0.97	−0.14	−0.08	0.06	99
Co	−0.95	−0.12	−0.01	0.10	98
Ni	−0.86	0.02	−0.14	0.28	89
Zn	0.05	−0.13	−0.25	0.84	87
As	−0.87	−0.27	0.00	0.22	95
Br	0.46	−0.09	0.67	−0.22	91
Rb	0.89	−0.17	0.23	−0.02	94
Sr	0.24	0.81	−0.13	−0.19	87
Sb	−0.62	−0.14	0.00	0.61	92
Cs	−0.94	−0.24	−0.01	0.13	99
Th	0.96	0.11	0.15	−0.05	99
U	0.91	0.22	0.10	−0.25	98
Cd	−0.14	0.55	−0.19	0.61	85
Pb	0.38	0.12	0.15	−0.72	85
Cu	−0.45	0.10	−0.66	0.32	82
Expl.Var, %	47	14	12	11	

**Table 4:** Values of the Contamination factor, and Pollution load index for the entire Republic of Moldova, Chisinau, and Balti.

Element	Background Values, mg/kg	Moldova	Chisinau	Balti
		CF		
Cu	9.4	0.9 ± 0.3 *	2.4	1.4
V	5.7	1.2 ± 0.7	2.5	3.2
Cr	5.5	1.3 ± 0.8	3.0	3.9
Fe	1820	1.5 ± 0.8	3.4	3.8
As	0.52	1.6 ± 0.7	3.7	3.9
Cd	0.26	0.5 ± 0.4	0.4	0.5
Zn	42	1.0 ± 0.3	1.1	1.4
Sb	0.15	1.6 ± 1.1	5.4	5.6
Pb	4.8	0.7 ± 0.3	1.1	1.4
U	0.13	1.8 ± 0.9	4.0	4.1
PLI		1.1 ± 0.4	2.4	2.3
PER		44.1		

*-mean± standard deviation.

## Data Availability

All data are presented in the manuscript.

## Supplementary Materials

The following are available online at https://www.mdpi.com/2223-7747/10/3/471/s1, Figure S1: Plot of eigenvalues, Table S1: Information about moss collection sites.

## Abbreviations

AAS	atomic absorption spectrometry
CF	contamination factor
CV	coefficients of variation
FA	factor analysis
MAD	Median Absolute Deviation
NAA	neutron activation analysis
PLI	Pollution load index
RI	Potential ecological risk index
SD	standard deviation
