# Supplementary material for: Accumulation of Potentially Toxic Elements in Mosses Collected in the Republic of Moldova
Source: Plants (Basel). 2021 Mar 2;10(3):471. doi: 10.3390/plants10030471 (PMC7999594; doi:10.3390/plants10030471)
Supplement: Supplementary file 1 [file plants-10-00471-s001.pdf]

Table S1 Information about moss collection sites

Sample number	Collection place	Latitude	Longitude
1	Riscani	47. 9709	27 .5453
2	Edinet	48. 1511	27 .2978
3	Falesti	48. 1849	27 .1367
4	Hlina	48. 2796	26 .8329
5	Briceni	48. 3567	27 .0357
6	Haradauti	48. 3624	27 .3078
7	Valcinet	48. 4438	27 .6985
8	Donduseni	48. 2644	27 .6070
9	Soroca	48. 1713	28 .2564
10	Glodeni	47. 7331	27 .5629
11	Falesti	47. 5869	27 .7008
12	Ungheni	47. 2164	27 .8192
13	Vulcanesti	47. 1435	28 .1468
14	Capriana	47. 0985	28 .4949
15	Calarasi	47. 2790	28 .2536
16	Cucioa	47. 4670	28 .1921
17	Balti	47. 7562	27 .8521
18	Singerei	47. 6312	28 .1775
19	Hirtop	47. 9002	28 .4487
20	Rezina	47. 7582	28 .9438
21	Orhei	47. 4023	28 .8239
22	Vadul lui Voda	47. 0775	29 .1023
23	Puhoi	46. 8295	29 .0765
24	Hirbovet	46. 8596	29 .3500
25	Causani	46. 6398	29 .3876
26	Stefan Voda	46. 5481	29 .6657
27	Troita	46. 5075	29 .0434
28	Comrat	46. 2614	28 .6490
29	Cahul	45. 9076	28 .1192
30	Tighec	46. 3674	28 .3845
31	Sarateni	46. 6015	28 .4439
32	Hincesti	46. 8125	28 .5640
33	Chisinau	47.0008	28 .8450
34	Scorteni	47.6355	28 .6667
35	Izvoare	47.9643	28 .1328
36	Drochia	48.0289	27 .7941
37	Decebal	48.2698	28 .0111
38	Slobozia Mare	45.6048	28 .1573

39	Baimaclia	46.1979	28 .3520
40	Valea Perjei	46.0133	28 .9391
41	Vinogradovka	45.7927	28 .5484
